# Personalised Advanced Therapies in Parkinson’s Disease: The Role of Non-Motor Symptoms Profile

**DOI:** 10.3390/jpm11080773

**Published:** 2021-08-07

**Authors:** Valentina Leta, Haidar S. Dafsari, Anna Sauerbier, Vinod Metta, Nataliya Titova, Lars Timmermann, Keyoumars Ashkan, Michael Samuel, Eero Pekkonen, Per Odin, Angelo Antonini, Pablo Martinez-Martin, Miriam Parry, Daniel J. van Wamelen, K. Ray Chaudhuri

**Affiliations:** 1 Department of Basic and Clinical Neurosciences, Institute of Psychiatry, Psychology & Neuroscience, King’s College London, London SE5 9RT, UK; valentina.1.leta@kcl.ac.uk (V.L.); anna.sauerbier@uk-koeln.de (A.S.); vinod.metta@nhs.net (V.M.); miriamparry@nhs.net (M.P.); daniel.van_wamelen@kcl.ac.uk (D.J.v.W.); 2 Parkinson’s Foundation Centre of Excellence, King’s College Hospital, London SE5 9RS, UK; m.samuel@nhs.net; 3 Department of Neurology, Faculty of Medicine, University of Cologne, 50937 Cologne, Germany; haidar.dafsari@uk-koeln.de; 4 Department of Neurology, Neurosurgery and Medical Genetics, Federal State Autonomous Educational Institution of Higher Education «N.I. Pirogov Russian National Research Medical University», Ministry of Health of the Russian Federation, 117997 Moscow, Russia; nattitova@yandex.ru; 5 Department of Neurodegenerative Diseases, Federal State Budgetary Institution «Federal Center of Brain and Neurotechnologies», Ministry of Health of the Russian Federation, 117997 Moscow, Russia; 6 Department of Neurology, University Hospital of Giessen and Marburg, Campus Marburg, 35043 Marburg, Hessen, Germany; lars.timmermann@uk-gm.de; 7 Neurosurgical Department, King’s College Hospital Foundation Trust, London SE5 9RS, UK; k.ashkan@nhs.net; 8 Department of Neurology, University of Helsinki, 00029 HUS Helsinki, Finland; eero.pekkonen@hus.fi; 9 Division of Neurology, Department of Clinical Sciences Lund, Lund University, P663+Q9 Lund, Sweden; per.odin@med.lu.se; 10 Parkinson and Movement Disorders Unit, Department of Neuroscience, University of Padua, 35138 Padua, Italy; angelo.antonini@unipd.it; 11 Centre for Networked Biomedical Research in Neurodegenerative Diseases (CIBERNED), Carlos III Institute of Health, 28031 Madrid, Spain; pmm650@hotmail.com; 12 Department of Neurology, Donders Institute for Brain, Cognition and Behaviour, Radboud University Medical Centre, 6500HB Nijmegen, The Netherlands

**Keywords:** Parkinson’s disease, device-aided therapies, non-motor symptoms, personalised medicine, apomorphine, levodopa-carbidopa intestinal gel, deep brain stimulation

## Abstract

Device-aided therapies, including levodopa-carbidopa intestinal gel infusion, apomorphine subcutaneous infusion, and deep brain stimulation, are available in many countries for the management of the advanced stage of Parkinson’s disease (PD). Currently, selection of device-aided therapies is mainly focused on patients’ motor profile while non-motor symptoms play a role limited to being regarded as possible exclusion criteria in the decision-making process for the delivery and sustenance of a successful treatment. Differential beneficial effects on specific non-motor symptoms of the currently available device-aided therapies for PD are emerging and these could hold relevant clinical implications. In this viewpoint, we suggest that specific non-motor symptoms could be used as an additional anchor to motor symptoms and not merely as exclusion criteria to deliver bespoke and patient-specific personalised therapy for advanced PD.

## 1. Advanced Parkinson’s Disease: The Clinical Scenario

Parkinson’s disease (PD) is a heterogenous syndromic disorder with a complex natural history, spanning prodromal to palliative stages [1,2]. While early motor phases of PD can be effectively managed by oral and transdermal dopamine replacement therapies, treatment of the more advanced phases remains a challenge, partly complicated by the requirement to choose which device-aided therapies (DAT) to offer to which patients, including levodopa-carbidopa intestinal gel infusion (LCIG) with or without entacapone, subcutaneous apomorphine infusion (APO), and deep brain stimulation (DBS). An optimal therapeutic choice is important as advanced PD is associated with motor and non-motor complications which may be refractory to standard oral/transdermal therapy negatively affecting quality of life [3,4,5,6]. International consensus and standard guidelines have attempted to address ideal DAT selection, but the latter still remains an unmet need [7,8,9]. A recent initiative based on an international Delphi-panel approach identified key motor, non-motor, and functional indicators of advanced PD [10], externally validated in the OBSERVE-PD study [11]. This has led to the development of the ‘5-2-1’ paradigm (≥5-times oral levodopa doses/day, ≥2 h of ‘off’ symptoms/day, ≥1 h of troublesome dyskinesia/day) to identify motor aspects of advanced PD and ensure timely referral for DAT initiation [10]. The interim analysis of DUOGLOBE, an observational study evaluating the long-term effectiveness of LCIG in patients with advanced PD, showed that only 20% of patients met all of the 5-2-1 criteria, but 98% met at least one criterion, highlighting the need for further refinement and personalisation of DAT selection [12].

A clinically relevant issue is the debate on whether earlier (than currently adopted in clinical practice) initiation of DAT may be beneficial for patients with PD. The EARLYSTIM study as well as the post-hoc analysis of the GLORIA registry have explored an earlier introduction of DBS and LCIG, respectively, but appropriate timing of DAT initiation largely remains an area of debate [13,14,15]. Moreover, older patients (≥75 years), for whom DBS is often not considered because of risk-benefit uncertainty, may nonetheless benefit from a modified approach involving DBS of several nuclei [16]. Another emergent debate is focused on how non-motor symptoms (NMS) may guide DAT selection for patients with PD as a positive inclusion criterion, rather than being used purely as an exclusion criterion, e.g., severe depression as a contraindication for DBS and severe hallucinations for APO.

Finally, also in relation to initiatives of providing earlier initiation of DAT in patients with PD, the relatively high costs of DAT need to be taken into account. Here, the societal impact of advanced PD is considerable as the 20% most affected patients are responsible for around 70% of secondary care costs [17]. The costs of DAT can be considerable, but NMS have not been taken into account in cost-effectiveness analyses [18]. This is a relevant observation as NMS contribute at least equally, if not more, to quality of life as motor symptoms [19,20]. Additionally, motor fluctuations, the most common indication for DAT, are often accompanied by non-motor fluctuations, adding to perceived quality of life [21,22]. Thus, it seems reasonable to include NMS in the decision to initiate DAT in patients with PD, especially for those with only moderate motor symptoms but severe non-motor burden [23].

Therefore, in this viewpoint, we will focus on the emerging role of the non-motor profile integral to the choice and outcomes of personalised medicine [1] when delivering DAT in PD. We aim to delineate the emerging field of non-motor indications for DAT and discuss possible implications for clinical practice.

## 2. Current Use of Non-Motor Symptoms in Device-Aided Therapies Selection

NMS have been proposed as criteria to consider for use of DAT; however, they are not considered in most country-based guidelines by licensing authorities or are merely used as exclusion criteria. The latter has been reviewed as part of the NAVIGATE PD initiative [7], for instance, and NMS constitute both relative and absolute contraindications for certain DAT while data suggests NMS could be improved by DAT. An absolute contraindication (in most countries) for all DAT is severe dementia, whereas non-motor aspects representing relative contraindications are more diverse. For APO and LCIG these include impulse control disorder and dopamine dysregulation syndrome, along with mild to moderate cognitive dysfunction; for DBS the main non-motor contraindications are severe depression and clinically relevant cognitive impairments [7,24]. Moreover, presence of symptomatic orthostatic hypotension, excessive daytime sleepiness, and severe hallucinations could be considered exclusion criteria for APO [25].

## 3. Device-Aided Therapies and Differential Effect on Non-Motor Symptoms

While therapeutic decisions and research on DAT have largely focused on the influence and effect on motor symptoms, NMS are an integral feature of PD and, therefore, should play an active part in the decision-making process to select the ideal DAT for patients with PD [7,10]. Although APO, LCIG and bilateral subthalamic nucleus (STN) DBS have been available for many years for the treatment of PD in many countries, head-to-head comparative studies are limited. Following on from the original EuroInf study [26], the EuroInf 2 study is the first and only study concurrently comparing all three DAT [27]. Although open-label in its design, it offers Class IIb evidence on the differential effects of these DAT on NMS measured by the NMS scale (NMSS) total burden and its domain’s scores. In agreement with other studies, all three therapeutic options confirmed an improvement in motor complications, Hoehn and Yahr stage and quality of life [26,27,28,29,30,31,32]. Although all three DAT decreased total NMS burden, interestingly, each treatment appeared to have a bias towards specific NMS thus providing some early indications of varied responsiveness to each therapy. For instance, in this cohort of 173 patients, APO decreased the attention/memory domain scores, while bilateral STN-DBS and LCIG did to a lesser extent which was not statically significant. Nonetheless, it needs to be acknowledged that patients with cognitive problems would be excluded a priori from receiving DBS. Similarly, in this study patients receiving APO had higher NMSS attention/memory baseline scores compared to the other groups, leaving more room for improvement. Data on patients with severe attention/memory problems are not available. On the other hand, DBS and LCIG appeared to reduce the urinary and gastrointestinal domains scores, respectively. All three treatment options decreased the mood/apathy and miscellaneous domains scores, the latter including weight changes, altered thermoregulation and olfaction as well as unexplained pain. Improvements here were heterogeneous, and while APO reduced weight change-related scores, LCIG and DBS improved most of the symptoms contained within the miscellaneous domain. Aspects of sleep dysfunction and fatigue as measured by the NMSS also improved with both LCIG and bilateral STN-DBS, but not after APO initiation. Finally, there is evidence to suggest that APO and bilateral STN-DBS decrease the perceptual problems and hallucinations domain scores, although typically these are considered contra-indications [10]. The mechanisms behind these associations need to be further elucidated; however, it is possible to argue that, for instance, historical presence of visual hallucinations which are mainly drug-induced, and which might subside after drug withdrawal at the expense of a troublesome motor worsening, might benefit from DAT initiation. Finally, combined DAT-related data is also emerging, and may help us to overcome specific issues [33,34,35,36,37,38].

### 3.1. Non-Motor Effects of Deep Brain Stimulation

Important conceptual advances may hold promise in relation to the delivery of personalised medicine and DAT in PD [2]. In addition to the abovementioned EuroInf studies, this is exemplified by several studies that have been conducted on the non-motor effects of DBS, showing improvements in several non-motor areas that have been reviewed elsewhere [39,40,41,42,43,44]. In brief, a recent meta-analysis, including 48 studies with mainly 12-month follow-up data, suggested post-STN-DBS improvements of depression and anxiety-related symptoms but increased apathy [41]. Another meta-analysis of seven studies with follow-up data ranging from three to 24 months showed post-STN-DBS improvements in sleep quality and restless leg syndrome; however, a high degree of heterogeneity among studies was reported [39,44,45], and few studies have investigated the effect of STN-DBS on REM sleep behaviour disorder [42,46]. Another recently published review summarised post-DBS positive outcomes related to urinary dysfunction (mean bladder volumes at desire and urge point to void), while controversial and limited data are available in relation to sexual, cardiovascular, thermoregulatory and gastrointestinal dysfunction [40]. Finally, even though presence of dementia is a contraindication for DBS, a systematic review of 13 studies showed that although there was a decline in verbal fluency and attention domains of cognition, other cognitive functions remained unchanged over a follow-up period ranging from six months to eight years [43]. It needs to be acknowledged that most included studies had small cohort sizes and heterogenous outcome measures.

Further advancements in relation to personalised medicine with DBS might be achieved by directing neurostimulation to specific parts of the basal ganglia and leveraging their specific connectivity profiles [47,48,49].

More theoretical approaches, such as adaptive DBS, have been developed as a method where DBS is turned on and off according to a closed-loop feedback signal recorded from the tissue surrounding the stimulating electrode. This may develop into personalised approach if it can show to activate DBS at times of necessity and reduce it at times of quiescence, for example in sleep, with the aim of a more physiological treatment and potentially reducing the frequency for battery replacements in non-rechargeable systems. Presently, limitations to the clinical application of adaptive DBS are: (1) Tremor frequency, beta-band and other oscillations required for the closed-loop feedback arc of adaptive DBS are not recordable in all patients with PD [50]; (2) beta-band activity represents not only pathological alterations, but is also modulated by physiological functions [51,52] (3) pathological tremor frequency and beta-band oscillations may, in some patients, reflect tremor, bradykinesia and rigidity, but not NMS [53]; (4) motor symptoms can fluctuate at different times during the course of the day than non-motor fluctuations [21,22]. As such, situations may arise in which the neurostimulation is not active because tremor frequency and beta-band oscillations cannot be detected, but the patient nonetheless presents with NMS such as pain or depressed mood. Therefore, studies are needed to investigate the effect of adaptive DBS on quality of life and NMS, not only motor symptoms [54].

### 3.2. Non-Motor Effects of Levodopa-Carbidopa Intestinal Gel Infusion

There is robust evidence on the effect of LCIG on NMS. In 2015, a systematic review identified eight open-label studies confirming that LCIG improved total NMS burden after a follow up period ranging from six to 25 months, with specific positive effects on sleep and autonomic dysfunction, and particularly gastrointestinal issues measured by the NMSS [55]. Additionally, more recent reviews have highlighted the non-motor effect of LCIG where a general improvement in the non-motor burden was noted [56,57]. Studies included in these reviews were, among others, the GLORIA registry, whose 24-month follow up data showed a remarkable beneficial effect of LCIG on sleep disturbances, apathy, and gastrointestinal dysfunction as measured by the NMSS [29], and the interim analysis of the DUOGLOBE study, where an overall improvement in the NMS total burden was also shown after only six months [12]. Additional open labels studies with 6-month follow-up data showed a post-LCIG improvement in NMS total burden, including reduction of the cardiovascular, attention/memory, urinary and miscellaneous domains scores of the NMSS [26,58]. Interestingly, the baseline total burden of NMS in PD can predict a robust total non-motor response to LCIG therapy at two years follow up. This observation can underpin DAT selection with an NMS focus, specifically when considering personalised LCIG therapy for instance [59].

### 3.3. Non-Motor Effects of Apomorphine Subcutaneous Infusion

Although APO has been in use longest compared with DBS and LCIG (APO became available on the European market in the early 1990s), data regarding APO and selection of this device-aided therapy based on patients’ non-motor profile is less obvious and the results from the double-blind TOLEDO study are awaited with interest [60]. However, several open-label and case report-based studies show that this treatment can have a beneficial effect on the NMS total burden as well as on specific non-motor areas, and these have been reviewed elsewhere [56,61,62]. In brief, there is evidence suggesting post-APO improvements in depression, anxiety, apathy, perceptual problems, cognitive impairment, sleep dysfunction (insomnia and restless leg syndrome), fatigue, urinary dysfunction (urinary frequency, urgency and nocturia), and gastrointestinal dysfunction (dribbling of saliva) as measured by the NMSS at both 6- and 12-month follow up [26,63]. The reported beneficial effect or tolerability of APO on mild visual hallucinations is of interest given that it is a dopamine D1 and D2 receptor agonist, and suggested underlying mechanisms include the associated reduction in oral medication and/or a psychotropic action of APO, possibly due to the piperidine moiety in its structure [64,65]. In addition, the potential beneficial role of APO on cerebral amyloid deposition is worth considering in relation to its positive modulatory effect on cognition [26,63,66,67].

## 4. Need for Personalised Treatment in Advanced Parkinson’s: Clinical Cases

**Figure 1:**
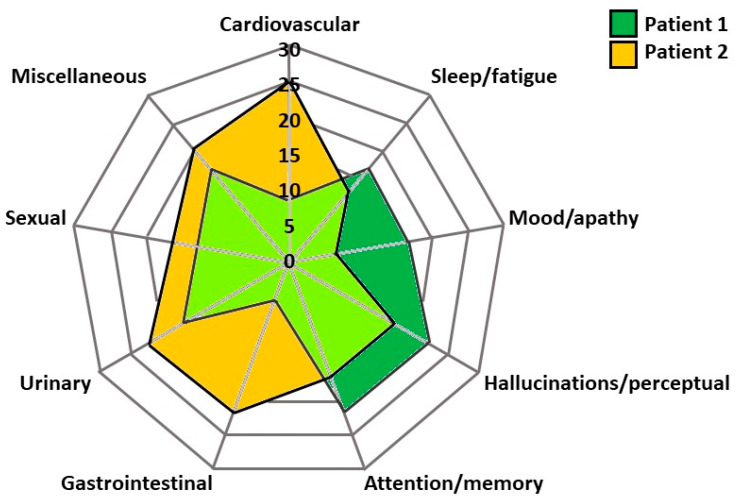
Radar chart of non-motor profile of two patients with advanced Parkinson’s disease assessed for initiation of device-aided treatments. The radar chart is based on the Non-Motor Symptoms Scale (NMSS) domains scores obtained as part of routine clinical assessment. While patient 1’s non-motor profile is dominated by mild attention/memory issues as well as mild perceptual problems, patient 2’s main complaint is dysautonomia, including cardiovascular, urinary and gastrointestinal dysfunction. Numbers represent the NMSS domains scores. The light green area represents the overlap in symptoms between the two patients.

Taking into account the distinct NMS effects of these three DAT, it can be postulated that the specific non-motor profile of patients with advanced PD may serve as an additional anchor to motor symptoms to deliver personalised medicine. Two illustrative clinical cases are presented in Figure 1 showing the different non-motor profile of two patients with advanced PD evaluated for DAT initiation.

The clinical assessment revealed that both patients suffered from motor complications including troublesome dyskinesia and motor fluctuations refractory to conventional therapies; in addition, the non-motor profile of patient 1 was dominated by mild cognitive decline and non-intrusive perceptual issues, whereas for patient 2, cardiovascular, urinary, and gastrointestinal dysfunction were particularly pronounced. On the basis of these two different non-motor profiles and according to the EuroInf 2 data, it can be argued that APO may represent the best therapeutic option for patient 1, while, for patient 2, APO may not be suitable as it may exacerbate pre-existing cardiovascular problems, including orthostatic hypotension. On the other hand, while LCIG may be useful to improve gastrointestinal symptoms, STN-DBS may be the best option to improve urinary dysfunction for patient 2. As such, it would be important to inquire which one of the two is the most troublesome/severe NMS to better tailor the decision-making process.

Other factors are also implicated in the delivery of personalised DAT in PD [2]. Evaluation of patient age, for instance, represents a key aspect in the assessment for DBS suitability; indeed, age >70 or 75 years is an exclusion criterion for DBS in some centres given the associated higher risk of complications [68]; nevertheless, biological age is more often taken into consideration than chronological age in addition to the fact that the impact of “healthy ageing” is growing [69]. Another relevant aspect of this decision-making process is the evaluation of comorbidities. For instance, poorly controlled diabetic patients with PD have a higher risk of developing skin infections and this should be considered in the evaluation for any DAT [70]. Other comorbidities, such as pre-existing significant and symptomatic peripheral neuropathy needs consideration for LCIG, impulse control disorder and intrusive psychosis (as opposed to mild non-intrusive psychosis) for APO, and severe depression or suicidal trends for DBS [7]. Last but not least, patient personality and preferences need to be taken into account: some active young patients may prefer a more invasive brain surgery than a percutaneous endoscopic gastrostomy in order to avoid carrying a visible infusion pump every day, and for a “quick fix” of dyskinesias and tremor [71]. Body weight has also emerged as an important aspect of the decision-making process [72]. Low body weight patients with advanced PD may develop pain, discomfort and worsening of postural problems with subsequent risk of falls when carrying a heavy infusion pump [73]. The advent of a smaller infusion pump with the new levodopa-carbidopa-entacapone intestinal gel product now licensed for use in Sweden and Germany may represent a significant advance in this respect [74,75]. Whether this new product will have an impact on NMS similar to LCIG remains unexplored. Evaluating the ability of the patient and/or caregiver to handle the medication and the device, as well as daily skin hygiene, is also critical [73].

## 5. Conclusions

**Figure 2:**
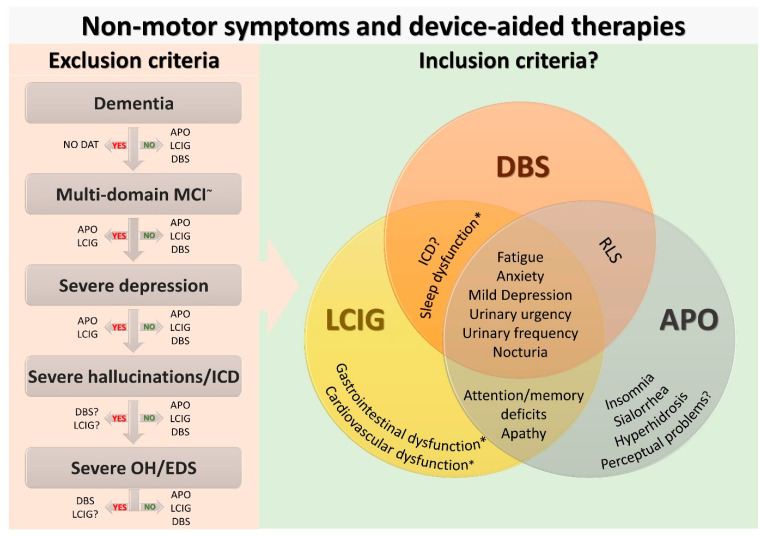
Non-motor enablers for a successful selection of device-aided therapy for patients with advanced Parkinson’s disease. The figure shows non-motor exclusion and possible inclusion criteria for a successful patient initiation on device-aided therapies. We emphasise that these conditions should not be considered an absolute contraindication or indication for the device-aided therapies and expert opinion based on multi-disciplinary assessments should have the final say. ^~^ Multi-domain MCI with a predominant cortical pattern (e.g., memory, language, visuospatial); * Further studies are needed to better clarify which aspect of sleep, gastrointestinal and cardiovascular dysfunction can improve after device-aided therapy initiation. Abbreviations: APO, apomorphine subcutaneous infusion; DAT, device-aided therapies; DBS, Deep brain stimulation; EDS, excessive daytime sleepiness; ICD, Impulse control disorder; LCIG, levodopa-carbidopa intestinal gel infusion; MCI; mild cognitive impairment; NMS, non-motors symptoms; OH, orthostatic hypotension; RLS, Restless legs syndrome.

Device-aided therapies are now established worldwide for the management of advanced Parkinson’s disease. While the emphasis of device-aided therapies selection remains based on the motor profile of patients with PD, non-motor symptoms have also been shown to play a part in the prognostic aspects of the successful delivery of these therapeutic options and are now included in the diagnostic algorithm of advanced PD. Considering the differential effect on non-motor symptoms of the currently available device-aided therapies, non-motor symptoms are relevant to delivering personalised medicine in Parkinson’s disease. We envisage that the identification of different motor and non-motor phenotypes of Parkinson’s may guide the delivery of personalised medicine in the advanced stage of the condition, perhaps guided by technology able to predict motor and non-motor responses to device-aided therapies on the basis of the patient-specific pre-intervention symptom’s profile. We suggest that non-motor symptoms are an important enabler of the constituents of the “circle of personalised medicine” and offers a chance to deliver bespoke personalised therapy for advanced PD (Figure 2).

## Data Availability

Not applicable.
